# Leukocytoclastic Vasculitis in a Patient With Rheumatoid Arthritis

**DOI:** 10.7759/cureus.17124

**Published:** 2021-08-12

**Authors:** Avijoy Roy Choudhury, Amba Roy Choudhury

**Affiliations:** 1 Internal Medicine, University of Western Australia, Perth, AUS; 2 General Practice, Mindarie Keys Medical Centre, Perth, AUS

**Keywords:** leukocytoclastic vasculitis, cutaneous vasculitis, small vessel vasculitis, corticosteroid treatment, rheumatoid arthritis

## Abstract

Leukocytoclastic vasculitis is a small vessel vasculitis that is usually confined to the skin with rare extracutaneous manifestation. While this condition can be idiopathic, it has been linked with systemic autoimmune conditions, malignancies, infections, and drugs. In this paper, we present a case of a patient who presented with leukocytoclastic vasculitis many years after her diagnosis of rheumatoid arthritis. It is important that physicians investigate leukocytoclastic vasculitis, as the condition, while often idiopathic, can be a presentation of something more sinister such as malignancy or systemic autoimmune condition.

## Introduction

Leukocytoclastic vasculitis is a small vessel vasculitis that is usually confined to the skin with rare manifestations extracutaneous. It is an immune complex-mediated process of the dermal capillaries and venules, which is characterized by histopathology [1]. Clinical presentation usually involves palpable purpura usually in the lower extremities [2]. Diagnosis is usually confirmed with a punch biopsy that is performed with direct immunofluorescence studies [3]. This case outlines a patient with leukocytoclastic vasculitis and neutropenia, who has a long-standing history of rheumatoid arthritis. This report aims to outline the importance of early treatment and management of both the condition and symptoms, which resulted in positive outcomes for the patient. Furthermore, this report also aims to emphasize the importance of investigating a patient with leukocytoclastic vasculitis due to its strong relationship with other conditions. 

## Case presentation

**Figure 1:**
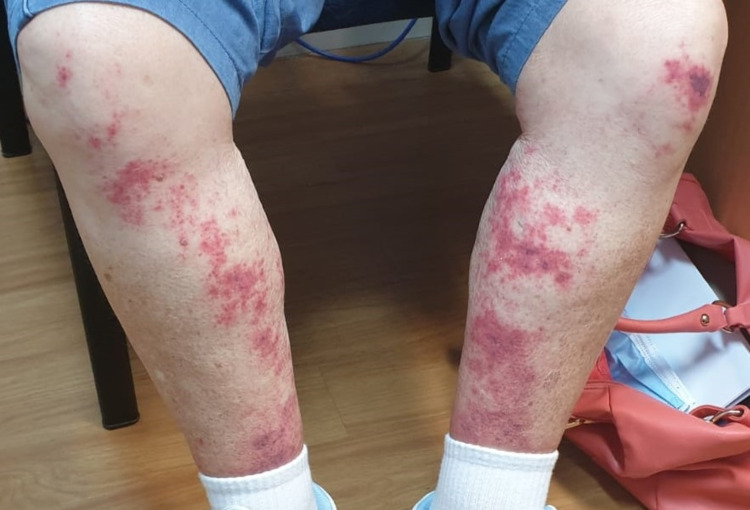
Bilateral shin rashes on initial presentation

A 71-year-old female presented to the clinic with rashes on bilateral shins and abdomen (Figure 1). The rashes were petechiae-like and pruritic. The patient appeared well with no systemic symptoms including no fever, no arthralgia, and no infective symptoms. No organomegaly was present. The patient has had no recent change in medication and denies any new clothes or gardening. The rash was unresponsive to antihistamines. The patient has a 25-year history of rheumatoid arthritis for which she has been taking 20 mg/week of methotrexate for the past 10 years. The patient has also been taking folic acid supplementation of 5 mg daily. 

Blood tests were ordered for the patient including full blood count (FBC), urea, electrolytes, and creatinine (UEC), erythrocyte sedimentation rate (ESR), folate levels, vitamin B12 levels, and liver function tests (LFTs). Results from the tests showed significant neutropenia with a neutrophil count of 0.8 x 109/L. The patient's folate level was greater than 54.0 μg/L and vitamin B12 level was 269 pg/mL, meaning both results were within normal limits. 

The patient returned two days later with the rashes having become more erythematous and extensive. The rashes were now present on both ears and on the left side of the face in addition to the shins and abdomen. The patient was sent to the hospital where during the admission she was given a stat dose of filgrastim and was commenced on prednisolone and discharged. Punch biopsies of rashes were taken to aid with diagnosis.

The patient returned to the clinic 25 days following the start of the prednisolone treatment and the rashes had disappeared and neutropenia had resolved. The patient was weaned off the prednisolone over four weeks to 0 mg and was diagnosed with leukocytoclastic vasculitis from biopsy results. The presentation of leukocytoclastic vasculitis was likely related to the patient’s rheumatoid arthritis.

## Discussion

Leukocytoclastic vasculitis has an annual incidence of approximately 45 million cases per year [1]. Leukocytoclastic vasculitis affects vessels including arterioles, capillaries, and postcapillary venules. The core histological features that make the diagnosis of leukocytoclastic vasculitis include fibrinoid necrosis and activation, death, and degranulation of neutrophils in the presence of neutrophilic infiltration within and around the vessel wall [4].

Almost half of all cases of leukocytoclastic vasculitis are idiopathic. Infections, malignancies, and drugs are also common triggers for the development of leukocytoclastic vasculitis. Systemic autoimmune conditions such as rheumatoid arthritis, systemic lupus erythematosus, and Sjogren’s syndrome also have a well-established connection with the development of leukocytoclastic vasculitis [1,5].

In patients with well-established rheumatoid arthritis and Sjogren’s syndrome, leukocytoclastic vasculitis is often a complication that arises during the course of the disease. The vasculitis can arise without any changes in the management of the systemic autoimmune condition. The presentation of leukocytoclastic vasculitis usually arises multiple years after established diagnosis. In relation to patients with systemic lupus erythematosus that develop leukocytoclastic vasculitis, the presentation is also usually many years following diagnosis, although there have been cases where it has been the first presentation of the systemic autoimmune condition [1,6].

It is important to also consider malignancy as a potential cause of leukocytoclastic vasculitis in this patient considering the patient's age. In patients over the age of 50, leukocytoclastic vasculitis is more likely to be linked to malignancy. Recognition of leukocytoclastic vasculitis as a marker for malignancy is associated with better outcomes and higher rates of survival [7].

Studies have shown that when leukocytoclastic vasculitis is caused by an underlying systemic autoimmune disease, better control of the disease may be the best method of treatment and ensuring the patient suffers from no relapses. Other methods for treatment include systemic corticosteroids and the use of compression stocking to reduce purpure [8].

While the prognosis is usually favorable for patients with leukocytoclastic vasculitis, it needs to be investigated and often needs to be treated as there is a risk of ulceration, which often needs the aid of agents such as corticosteroids to resolve [1]. Furthermore, the presentation of leukocytoclastic vasculitis may often indicate systemic illness such as malignancies or systemic autoimmune conditions [4].

In relation to the patient's neutropenia, it is unlikely to be caused by methotrexate as the patient had been taking the medication over a long period of time and folate levels were within normal limits [9]. Although splenomegaly was not present during the presentation, the neutropenia could potentially have been a result of Felty's syndrome, considering the patient's longstanding rheumatoid arthritis. Furthermore, there is evidence suggesting Felty's syndrome may be asymptomatic supporting the possibility of it being the cause of the patient's neutropenia [10]. 

## Conclusions

We have reported a case of a 71-year-old female who presented with leukocytoclastic vasculitis with a related history of rheumatoid arthritis. The patient responded well to treatment with corticosteroids and symptoms resolved. The prognosis of the condition is generally good, but it needs to be investigated to ensure there is no underlying malignancy, infection, or autoimmune condition as they are strongly associated with leukocytoclastic vasculitis.
